# Digestive System Involvement During Coronavirus Disease 2019; the Newest Clinical Features and Potential Mechanisms: Digestive System Involvements and COVID-19

**DOI:** 10.31661/gmj.v11i.2569

**Published:** 2022-12-31

**Authors:** Aida Najafi Kashkooli, Parisa Jooya, Farzaneh Navari, Neda Gorjizadeh, Maryam Poudineh, Neda Pouralimohamadi, Asma Asadian, Hamidreza Sabet

**Affiliations:** ^1^ Anesthesiology and Critical Care Research Center, Shiraz University of Medical Sciences, Shiraz, Iran; ^2^ Department of Family Medicine, School of Medicine, Shiraz University of Medical Sciences, Shiraz, Iran; ^3^ Torbat Heydarieh, University of Medical Sciences, Torbat Heydarieh, Iran; ^4^ Department of Gastroenterology, Tehran University of Medical Sciences, Tehran, Iran; ^5^ School of Medicine, Mashhad Azad University, Mashhad, Iran; ^6^ Department of Medical Journalism, Faculty of Paramedical Sciences, Shiraz University of Medical Sciences, Shiraz, Iran

**Keywords:** COVID-19, Digestive System, Gastrointestinal Tract, Liver Injury, Pancreas

## Abstract

The coronavirus disease 2019 (COVID-19), which is caused by the severe acute respiratory syndrome coronavirus-2 (SARS-CoV-2), has been recognized as a worldwide pandemic and mostly affects the respiratory system. A considerable proportion of patients; however, might also experience gastrointestinal (GI) manifestations. Several investigations have assessed GI and hepatic involvement in this disease, although the mechanisms of these involvements in relation to the progression of COVID-19 remain unclear. This review summarized the clinical observations and the main mechanisms behind GI, liver, and pancreatic involvement among COVID-19 patients.

## Introduction

The coronavirus disease 2019 (COVID-19), caused by the severe acute respiratory syndrome coronavirus-2 (SARS-CoV-2), has now become a worldwide pandemic [1][2]. Although SARS-CoV-2 is primarily a respiratory pathogen, systemic organ involvement has been reported in many cases. Numerous studies have indicated a close relationship between gastrointestinal (GI) damage and COVID-19 [3]. GI manifestations, including vomiting, diarrhea, nausea, and loss of appetite are common among COVID-19-infected patients [3]. Also, hepatic abnormalities characterized by the elevation of liver enzymes and albumin reduction were observed among these patients [3]. The correlation between the severity of COVID-19 and GI and hepatic involvement during COVID-19 has been frequently demonstrated. Angiotensin-converting enzyme 2 (ACE2), which acts as the SARS-CoV-2 receptor, is highly expressed in most areas of the digestive system organs, including the GI tract, liver, and pancreas, allowing for further damage [4]. Understanding the exact mechanisms of digestive system injuries are important for disease management and designing new therapeutic approaches. This review provides comprehensive information regarding the characteristics of GI, liver, and pancreatic involvement during COVID-19 and discusses the potential processes through which SARS-CoV-2 exerts its harmful effects on the digestive system.

## 1. GI Involvement

**Figure-1:**
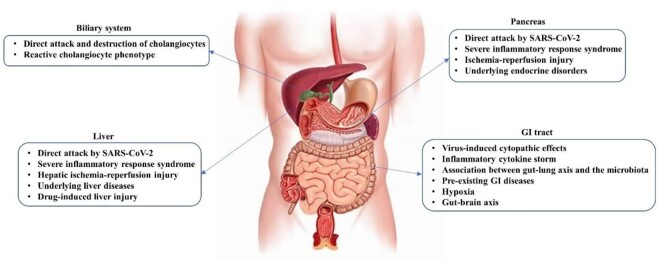
Figure 1. The potential mechanisms of COVID-19-associated GI, liver, biliary system, and pancreas injuries.

**Table:** Table 1. The Most Prevalent GI Symptoms Reported in Systematic Reviews and Meta-Analyses.

**Authors**	**Year**	**Age (year)**	**Sex (male%)**	**No. of studies included**	**No. of patients included **	**The most common GI symptoms **
**Mao et al. [6] **	2020	NM	NM	35	6686	Diarrhea, nausea/vomiting, and lost appetite
**Cheung et al. [10] **	2020	45.1	57.3	60	4243	Diarrhea, nausea, vomiting, and abdominal discomfort
**Han Cha et al. [12] **	2020	NM	NM	NM	NM	Diarrhea, nausea, vomiting, and abdominal discomfort
**Aziz et al. [13] **	2020	43.5	51.8	83	26912	Diarrhea
**Parasa et al. [14] **	2020	52.2	66.8	29	4805	Diarrhea and nausea/vomiting
**Dong et al. [15] **	2021	36-62	55	31	4682	Diarrhea and anorexia
**Zarifian et al. [16] **	2021	NM	47	67	13251	Anorexia, diarrhea, and nausea
**Suresh Kumar et al. [17] **	2020	NM	NM	17	2477	Diarrhea and nausea/vomiting
**Tariq et al. [18] **	2020	1-96	61.6	78	12797	Diarrhea, nausea, vomiting, and abdominal pain
**Rokkas et al. [19] **	2020	NM	NM	37	5601	Diarrhea, nausea, vomiting, and abdominal pain
**Li et al. [20] **	2020	46.7	51.8	212	281461	Diarrhea, nausea, vomiting, and abdominal pain
**Ashish Kumar et al. [21] **	2020	48.7	54	62	8301	Diarrhea, nausea, vomiting, and abdominal pain
**Shehab et al. [22] **	2021	55.6	45.2	158	78798	Diarrhea and nausea
**Merola et al. [23] **	2020	NM	NM	33	4434	Diarrhea, nausea, poor appetite, and abdominal pain
**Dorrell et al. [24] **	2020	NM	NM	108	17776	Diarrhea, nausea, vomiting, and abdominal pain
**Wang et al. [25] **	2020	NM	NM	21	3024	Diarrhea, nausea, and abdominal pain
**Sultan et al. [26] **	2020	NM	NM	47	10890	Diarrhea, nausea, and abdominal pain

**GI:** Gastrointestinal; **NM:** Not mentioned

### 1.1. COVID-19 and GI Clinical Features

Although the most prevalent signs of COVID-19 are the ones emanating from the respiratory tract, the data suggest several additional GI tract symptoms, namely diarrhea, nausea, vomiting, anorexia, GI bleeding, and stomach discomfort [5]. Even though some meta-analyses and reviews reported the prevailing GI symptoms in COVID-19 patients, the exact prevalence of GI injuries remains a matter of debate. Mao *et al*. showed the prevalence of digestive symptoms in 15% of the infected patients, out of which nausea and vomiting were the most common, followed by diarrhea and anorexia [6]. Notably, approximately 10% of the patients showed GI symptoms without respiratory manifestations at admission. As a result, they were more prone to have a late disease diagnosis, which led to considerable difficulties and rendered them a source of virus spread [6][7]. While anorexia was observed as the most common (39.9%-50.2%) symptom of the disease in one study, diarrhea was reported by other studies as the most prevalent (2%-50%) sign in both adult and pediatric populations [8][9]. Another review reported that GI symptoms accounted for 17.6% of COVID-19 manifestations [10]. GI symptoms are believed to develop in about one-fifth of the patients. GI symptoms are normally exacerbated as the disease progresses, denoting a more insidious disease incidence [11]. Also, patients with COVID-19 experience vomiting, stomach discomfort, and GI bleeding, albeit on small scales [11]. Regarding Table-1, the most common clinical GI symptoms in patients with COVID-19 were diarrhea, nausea, vomiting, anorexia, and stomach pain/discomfort, as reported in most published systematic reviews and meta-analyses [12][13][14][15][16][17][18][19][20][21][22][23][24][25][26].

### 1.2. Possible Mechanisms Involved in GI Track During COVID-19

#### 1.2.1 Direct Attack by SARS-COV-2

Possible mechanisms involved in the digestive system during COVID-19 are illustrated in Figure-1. SARS-CoV-2 can enter the cell and replicate via attachment to the ACE2 receptor of the cells. Although ACE2 is shown to be expressed in type-2 alveolar cells, its expression abounds in the epithelial cells of the GI system [27]. The induction of GI symptoms by SARS-CoV-2 has not been thoroughly investigated. For CoV infection to occur, the virus first needs to gain entry to the host cells. Similar to SARS-CoV, SARS-CoV-2 penetrates the host cells through the viral receptor ACE2 [8]. The presence of the viral nucleocapsid protein in COVID-19 patients has been confirmed in nearly all of the GI lumen, including duodenal, rectal glandular, and gastric epithelial cells, except for the esophagus [27]. Liang *et al*. investigated ACE2 expression and dispersion in a number of cell types as well as human tissues and suggested that the small intestine exhibited a high expression of ACE2, particularly in distal and proximal enterocytes [28].

Moreover, Zhang *et al*. used bioinformatics investigations to examine single-cell transcriptome and proportion in five public datasets comprising single-cell transcriptomes of the colon, ileum, gastric, esophagus, and lung [29]. It was shown that the expression of ACE2 was high in the stratified upper epithelial cells of the esophagus, which can account for the existence of SARS-CoV-2 in esophageal erosion [30]. Another finding was the higher expression of ACE2 in the absorptive enterocytes of the colon and ileum than in the lung [29]. Furthermore, SARS-CoV-2 entry into the host cell depends on the cellular serine protease, i.e., transmembrane protease serine 2 (TMPRSS2), which separates the S protein of the virus on the cell membrane [31]. The ACE2 receptor and TMPRSS2 are required to blend cellular and viral membranes [31]. It was found that TMPRSS2 and ACE2 were not only expressed simultaneously in esophageal upper epithelial, gland cells, and lung alveolar type 2 cells but also were strongly expressed in the colon and the ileum. This finding implies that the virus can penetrate the digestive tract enterocytes. It is unclear whether intestinal inflammation increases the expression of ACE2 in the gut and puts patients suffering from inflammatory bowel disease (IBD) at higher risk [32]. The virus nucleocapsid protein was observed in the duodenal, gastric, and rectal glandular cytoplasm, albeit not in the epithelial cells of the esophagus [8][27]. This signifies that SARS-CoV-2 might attack GI cells, particularly the stomach and intestine epithelial cells. It could be assumed that the precise mechanism through which SARS-CoV-2 interacts with the GI tract has yet to be discovered. Nevertheless, whether the available evidence suggests that the GI manifestations in COVID-19 are created by SARS-CoV-2 attacking the GI tract directly remains a key question.

### 1.2.2. Inflammatory Cytokine Storm Mediated by the Immune System

It is thought that SARS-CoV-2 infection disturbs inflammatory cytokine function in patients with COVID-19 [33]. Various reports also revealed that COVID-19 severity is associated with cytokine rates in patients [33][34]. Additionally, patients with COVID-19 who have pre-existing comorbidities, namely diabetes, hypertension, obesity, cardiovascular disorders, and asthma, as well as those who are elderly, are more vulnerable to inflammation [35][36]. Also, cytokine storm has a correlation with the development of acute respiratory distress syndrome (ARDS) and the poor functioning of multiple organs outside the lung during the COVID-19 progress [37][38]. Cytokine storms can lead to heightened COVID-19 conditions among patients with GI diseases [33].

Research reported that SARS-CoV-2 swiftly stimulates T cell activation and results in the release of various inflammatory cytokines, e.g., interleukin (IL)-1, IL-6, granulocyte-macrophage colony-stimulating factor (GM-CSF), monocyte chemoattractant protein-1 (MCP-1), and interferon gamma (IFN-γ). GM-CSF stimulates CD16^+^ and CD14^+^ cells, as well as monocytes, as a result of which inflammatory cytokine levels are increased, exacerbating the inflammatory cascade [39]. This heightened immune reaction induces tissue damage. In COVID-19 infection, T cells from the peripheral blood lead to more significant cytotoxic function with more cytotoxic granules, perforin, and granulysin, suggesting that stimulated T cells might accelerate systemic inflammation [40]. Furthermore, cells that express ACE2 produce several pro-inflammatory cytokines, including tumor necrosis factor-alpha (TNF-α), MCP-1, IL-6, IL-1, and tumor growth factor [32]. Distinctly, murine models of COVID-19 displayed a scarcity of ACE2 in the colon, resulting in increased vulnerability to inflammation and colitis developed owing to the diminished antimicrobial peptides and the change in gut microbiota, which eventually led to diarrhea. Nonetheless, this mechanism requires further investigation in humans [41].

### 1.2.3. Pathogenic Connections Between the Gut-lung Axis and the Microbiota in COVID-19

The function and composition of the respiratory tract flora influence the GI tract via the immune system. Similarly, the imbalance of GI flora also impacts the respiratory tract microbiota through the same mechanism, indicating a remarkable association between both mucosal compartments. This reciprocal effect is called the gut-lung axis [42]. Moreover, the imbalance of intestinal flora is related to the increased mortality rate in other respiratory infections mainly due to decreased anti-inflammatory/regulatory mechanisms and deteriorated inflammation in the gut and lung [43].

Several studies demonstrated a relationship between changes in intestinal microecology and respiratory viral infections [44][45]. In this regard, studies have shown that ACE2 can modulate intestinal microbiota homeostasis via amino acids [45]. Microbiota might ferment into short-chain fatty acids (SCFAs). Even though most SCFAs are metabolized, the unmetabolized ones increase naive CD4+ T cells in the bloodstream. CD4^+^ T cells are crucial in chronic enteritis and mucosal immunity. To penetrate the small intestine, CD4^+^ T cells require C-C chemokine receptor type 9 (CCR9) [46]. This finding was confirmed by Wang *et al*. that reported increased levels of lung-derived CCR9 CD4^+^T cells after viral infections [46]. CCL25, expressed by the small intestinal epithelium via recruiting the CCR9 CD4^+^ T cells into the small intestine, contributes to immune disturbance and exacerbates the gut flora homeostasis [47][48]. Afterward, the intestinal microbiota damage drives the increased Th17 cells into the small intestine. It also produces abundant IL-17A, leading to neutrophil recruitment, which causes GI symptoms [49]. Subsequently, bacteria and cytokines move toward the lung through the blood circulation, impacting the lung immune system as well as inflammation [45]. This bilateral interaction describes the gut-lung axis theory.

In summary, due to inflammation and dysbiosis and given that SARS-CoV-2 influences the mucous membranes of the GI and respiratory tracts, it is speculated that adjunctive therapies focusing on re-stabilizing eubiosis and modulating intestine microbiota might be critical therapeutic approaches to prevent and decrease COVID-19 complications.

### 1.2.4. Patients with Pre-existing GI Diseases

COVID-19 could influence the body through pre-existing GI conditions, and IBD, such as ulcerative colitis and Crohn's disease, is a great concern. It is established that these conditions influence the prognosis of COVID-19 patients [50].

A population-based investigation by Maconi *et al*. compared the risk of COVID-19 in IBD patients and control subjects [51]. Their findings revealed that IBD patients do not have a higher risk of COVID-19-specific manifestations or more severe disease than gastroenterology patients [51]. On the other hand, patients with persistent IBD are shown to be part of the COVID-19 high-risk population [52]. IBD patients are more likely to be infected mainly because of the fragile nature of their ileum and terminal colon [52]. Furthermore, the expression of ACE2 protein is upregulated at the sites of inflammation. In fact, most cases of IBD are treated through immunotherapy, thereby affecting the body’s reaction to pathogen resistance and increasing the risk of infection [53][54]. Consequently, patients with IBD become more vulnerable to COVID-19 pneumonia concerning viral receptors and immunotherapy; however, it is noteworthy that no conclusive evidence has been found as of yet.

### 1.2.5. Other Possible Mechanisms

In addition to inflammation, pre-existing GI diseases, the gut-lung axis, and dysfunction of ACE2, some other mechanisms are potentially implicated in the development of GI symptoms in COVID-19 patients [55]. Considering that hypoxia is an important clinical indication in patients with COVID-19 and has a critical role in intestinal homeostasis, which includes microbiota function and composition, oxygen deprivation is of great significance in GI disorders as well as disease severity [56]. Findings revealed that SARS-CoV-2 possibly influences the central nervous system [57]. As the gut-brain axis is of utmost significance, it is assumed that it has a critical role in GI disorders during SARS-CoV-2 infection. Moreover, SARS-CoV-2 could impact the enteric nervous system either directly through viral infection or an evoked immunological response (e.g., inflammatory cytokines), diarrhea is increased, and the vagus nerve is stimulated to increase vomiting [58]. In addition to what has been previously mentioned, given that these GI manifestations are not specific to COVID-19 and might be observed in patients without this condition, namely patients with IBD, peptic ulcer disease, and other GI infections, as well as patients taking antibiotics, proton-pump inhibitors, non-steroidal anti-inflammatory drugs, traditional remedies, and similar treatments, it is essential that physicians gain a thorough understanding before suspecting COVID-19, especially in cases where patients have pre-existing GI disorders [59].

## 2. Liver Involvement

### 2.1. Characteristics of Liver Injury During COVID-19

The occurrence of several organ failures, including acute heart failure, defective taste and smell, renal malfunction, skin disorders, and multiple organ dysfunction, demonstrates that SARS-CoV-2 infection is a systemic disease [60]. The liver is a vital organ and the impairment of its function enhances COVID-19 severity, worsens the prognosis, prolongs the hospital stay, and increases the risk of mortality [60][61].

Evidence of liver injury is reported based on abnormalities in biochemical markers, such as increased levels of alanine aminotransferase, aspartate aminotransferase, alkaline phosphatase (ALP), gamma-glutamyl transferase, and total bilirubin [62][63] as well as hypoproteinemia and prolonged prothrombin time [64][65][66]. In many clinical studies, COVID-19 was associated with vascular changes, such as dysregulation of intrahepatic portal vein branches, ductular proliferation, mild lobular and portal inflammation, hepatic cell necrosis, hepatic steatosis, and Kupffer cell activation [67]. Evidence of cholangiocellular injury was observed in COVID-19 patients, demonstrated by increased plasma γGT and ALP, bile plug formation, and bile duct proliferation [67][68][69].

Several previous investigations showed that liver dysfunction or injury is reflected mainly by abnormal hepatic tests and pathologic findings. Here, we have comprehensively reviewed the underlying mechanisms behind liver involvement in COVID-19 patients. These mechanisms are discussed in follow.

### 2.2. Possible Mechanisms Involved in Liver Injury During COVID-19

#### 2.2.1. Direct Attack by SARS-CoV-2

As mentioned previously, the key receptor for SARS-CoV-2 entry into cells is ACE2 in conjunction with TMPRSS2 [70], both found on the surface of hepatocytes and cholangiocytes. Also, RNA of SARS-CoV-2 was observed in the liver tissue using real-time polymerase chain reaction [71]. Intact SARS-CoV-2 has been found in the cytoplasm of hepatocytes by electron microscopy. Other parts of the liver, including endothelial cells of the portal vein and vessel lumens can be infected by SARS-CoV-2, as demonstrated by in situ hybridization [72]. SARS-CoV-2 may impair liver function by direct cytopathy through cell lysis or the induction of apoptosis and necrosis of hepatocytes and cholangiocytes [73]. After the attachment of SARS-CoV-2 to its receptors on hepatocytes and cholangiocytes, its genome is released and replicated in vesicles containing the replicase-transcriptase complex [74]. SARS-CoV accessory and structural proteins are produced in host cells and assembled with viral RNA to form the mature virus. The virus destroys hepatocytes and cholangiocytes and infects neighboring cells [74].

Several other receptors, which have received less attention, are reported in the direct cytopathy of SARS-CoV-2. These include basigin (CD147 or BSG) and liver/lymph node-specific intercellular adhesion molecule-3-grabbing integrin (L-SIGN or CD209L) [75]. Different domains within a single S protein can interact with multiple receptors because the S glycoprotein is one of the largest viral spike glycoproteins. L-SIGN is a Ca^2+^-dependent lectin found in the endothelial tissue of the liver and lymph nodes. Studies demonstrated that human L-SIGN could serve as a portal of entry to hepatocytes for infectious SARS-CoV-2 [75]. CD147 is a transmembrane glycoprotein that regulates tumor cell migration, apoptosis, cell proliferation, and bacterial and viral infection [76]. Previous studies discovered that CD147 could interact with the spike protein of SARS-CoV-2. Amplification of SARS-CoV-2 was reported to be inhibited by the loss of CD147 or the blockage of CD147 using meplazumab, an anti-CD147 antibody. In non-susceptible BHK-21 cells, CD147 expression permits virus entry into the cells [76].

A growing body of evidence, namely the expression of SARS-CoV-2 receptors on the surface of hepatocytes and cholangiocytes, direct detection of the intact virus in the liver, and the presence of SARS-CoV-2 RNA in the liver tissue, suggest that at least part of the liver damage process can be explained by direct virus attack.

### 2.2.2. Severe Inflammatory Response Syndrome

Another pathological pathway leading to liver dysfunction during COVID-19 is dysregulated immune responses and hyperinflammation. The severe inflammatory response was demonstrated by an increase in inflammatory indicators, namely C-reactive protein, IL-6, IL-2, D-dimers, lactate dehydrogenase, and ferritin [77]. Inflammatory pathways start with SARS-CoV-2 replication in target cells, leading to cell death and the release of inflammation-inducing factors, including DNA, an apoptosis-associated speck-like protein with a caspase recruitment domain oligomers, and ATP as well as pro-inflammatory chemokines and cytokines, such as C-C motif chemokine ligand (CCL) 7, CCL2, GM-CSF, and IL-1b [78]. B and T cell immune response activation after these inflammatory signals recruits monocytes and macrophages to the infection site [79]. Simultaneously, activation of natural killer and T cells produces cytokines, including GM-CSF, TNF-α, and IFN-γ, and activates monocyte-derived macrophages [80]. The release of inflammatory factors such as IL-6 leads to a severe inflammatory response and causes considerable damage to the target tissues [81].

Hepatocytes infected with SARS-CoV-2 overexpress pro-inflammatory cytokines. The severe inflammatory response in the liver could upregulate the expression of ACE2 [82]. Therefore, it paves the way for virus attack and indirectly causes tissue destruction [82]. SARS-CoV-2-infected cholangiocytes also upregulate the expression of pro-inflammatory cytokines and induce a reactive cholangiocyte phenotype [82]. The reactive cholangiocyte phenotype leads to inflammation and fibrosis propagation [83]. One of the main cellular causes of fibrosis is hepatic stellate cell activation [83]. The severe inflammatory response created by SARS-CoV-2-associated cholangiocellular and hepatocellular damage can activate hepatic stellate cells and initiate fibrosis [84]. Indirect involvement of systemic inflammation can also activate Kupffer cells. Kupffer cells do not express ACE2; however, via the propagation of inflammatory response, they can promote liver injury by the recruitment of monocytes [85]. Propagation of inflammatory stimuli and overexpression of IL-6 and IL-1 can stimulate hepatocellular cholestasis via downregulating hepatobiliary uptake and excretory systems [85].

Recent information gives new insights into the effects of SARS-CoV-2-mediated severe inflammatory response on liver tissue damage and highlights the importance of anti-inflammatory interventions at the beginning of the infection.

### 2.2.3. Hepatic Ischemia-Reperfusion Injury

Hepatic ischemia-reperfusion damage is one of the probable mechanisms of liver tissue damage during COVID-19. This process consists of two steps: ischemia, which induces tissue damage, and the consequent reperfusion, which leads to inflammatory response [86]. The most important causes of hypoxia during COVID-19 are sepsis, respiratory failure, cardiac failure, right-sided heart failure, coagulopathy, and thrombosis [87]. Ischemia causes a lack of oxygen supply, resulting in hepatocyte death through ATP reduction, glycogen consumption, and disruption of lipid metabolism [88]. Vascular disorder-induced sinusoidal endothelial cell damage can further exacerbate ischemia. Destruction of the biliary epithelium is another consequence of hypoxic conditions [89]. All the aforementioned disorders lead to the production of many cell death-derived products. The reperfusion step after hypoxia aggravates the situation by releasing these products and activating the inflammatory response. Activation of platelets, neutrophils, and Kupffer cells causes a sequence of damaging cellular reactions and cell injury [88].

Furthermore, the ischemia-reperfusion injury disrupts the normal function of lymphatic vessels. These vessels delay the progression of COVID-19 through exudating cell debris, inflammatory markers, immune cells, and the virus. Hence, abnormal function of lymphatic vessels can lead to more damage [90].

Although the ischemia-reperfusion condition can contribute to liver injury, it is more important in critically ill patients admitted to the intensive care unit. In the majority of infected individuals, compensatory mechanisms provide an adequate oxygen supply for the tissue [91].

### 2.2.4. Underlying Liver Diseases

Patients with chronic liver diseases are known to be more susceptible to severe COVID-19 infection and have higher mortality [92]. Underlying liver diseases can exacerbate COVID-19-induced liver damage in different ways. Underlying liver diseases include chronic and acute conditions such as viral hepatitis, cancers, hemochromatosis, non-alcoholic fatty liver disease, alcoholic liver disease, primary biliary cholangitis, primary sclerosing cholangitis, autoimmune hepatitis, liver transplants, and some other less common diseases [93]. Nevertheless, we need to evaluate each condition separately to explain the precise mechanisms through that SARS-CoV-2 aggravates the liver injury. There are little data available regarding the effects of these different conditions on worsening COVID-19-induced liver damage. Some studies reported that low lymphocytes and platelets are common features in patients with severe liver diseases caused by cirrhosis-related immune dysfunction [92]. Elevated inflammatory response during chronic liver diseases is another possible mechanism explaining increased liver damage in these conditions [94]. The probable effect of prescribed drugs or other interventions should also be explored in these patients. Consequently, underlying liver diseases can synergize with COVID-19-induced liver damage, as demonstrated by more severe outcomes in patients with underlying liver disease. Therefore, strict precautions against COVID-19 should be taken in this group of patients.

### 2.2.5. Drug-related Liver Damage

It was suggested that drugs might induce liver damage during COVID-19. Several drugs are usually prescribed for SARS-CoV-2-infected patients, including remdesivir, ribavirin, lopinavir/ritonavir, oseltamivir, chloroquine/hydroxychloroquine, tocilizumab, methylprednisolone, arbidol, baricitinib, camostat, ribavirin, anticoagulants, acetaminophen, and azithromycin [95]. The liver metabolizes almost all of these medications, and a hepatotoxic potential has already been confirmed for most of them. Corticosteroids are among the most common drugs for COVID-19-infected patients. Steatosis and glycogenosis were reported during the administration of corticosteroids [96]. Tocilizumab exhibits hepatotoxic effects through interference with IL-6 signaling, an important regulator of hepatic regeneration. Acetaminophen exerts a direct hepatotoxic effect and can lead to severe liver damage and even mortality from acute liver failure [96]. Remdesivir, favipiravir, arbidol, and their metabolites have cytotoxic and mitochondrial toxic effects on the hepatocytes, and rapid elevations of aminotransferase were reported in their users [97]. Although ribavirin has not been associated with clinically apparent liver injury, it leads to hemolysis, which can cause tissue hypoxia [98]. Camostat and tocilizumab administration elevates the risk of liver damage and jaundice [99].

Simultaneous prescription of multiple drugs is frequent in COVID-19-infected individuals. Many drugs complete their metabolism in the liver, and drug-related liver damage is not unexpected. Hence, SARS-CoV-2 could exert direct and indirect deteriorating impacts on liver tissue, and the co-occurrence of these destructive effects with the drugs’ adverse effects leads to irreparable consequences.

## 3. Pancreatic Involvement

### 3.1. Characteristics of Pancreas Injury During COVID-19

Acute pancreatitis is the most common cause of GI hospitalization in the United States [14]. Previous studies reported that acute abdominal discomfort is associated with acute pancreatitis [14]. A three-fold increase in serum levels of lipase and/or amylase over the upper limit of normal has been observed. Several studies described metabolic abnormalities, including hyperglycemia, the elevation of HbA1c, ketoacidosis, the occurrence of new-onset diabetes, and insulin-deficient forms of diabetes [100]. Obesity and type 2 diabetes are linked to COVID-19 severity and mortality. The disease severity is minimized by glycemic control, which shows the direct correlation between COVID-19 severity and normal pancreatic function [100]. These results show that COVID-19-induced metabolic changes could be due to the harmful effects of the disease on pancreatic tissue.

Also, COVID-19 is associated with fibrosis, microthrombi, and pancreatic endotheliitis [101]. Other significant morphological and functional changes are increased levels of bihormonal insulin/glucagon-positive cells, impaired secretion of insulin, loss of insulin gene transcription, and lower levels of insulin-secretory granules in β-cells [102]. Considering all this information, it can be concluded that COVID-19 leads to evident pancreatic damage and interferes with normal endocrine and exocrine pancreatic functions.

### 3.2. Probable Mechanisms of Pancreatic Injury During COVID-19

Similar to liver damage, the direct attack of SARS-CoV-2 might lead to acute pancreatitis in patients with severe COVID-19. Cells from both the endocrine and exocrine parts of the pancreas express ACE2 receptors, which facilitate SARS-CoV-2 entrance into the host cells [103]. TMPRSS2, which is essential for SARS-CoV-2 entry, is also found in endocrine α- and β-cells, exocrine acinar and ductal cells, and vasculature endothelial cells [101]. Immunofluorescence studies have proved the existence of SARS-CoV-2 nucleoprotein in pancreatic ductal and endothelial cells. In patients with pre-existing type 2 diabetes, failure of insulin-producing β-cells aggravates the pathophysiology of impaired β-cell function [101]. Entry of SARS-CoV-2 into different pancreatic cells leads to the proliferation of the virus and cell death. The consequences depend on the type of infected cells. In the endocrine part, α-cells produce glucagon, β-cells secrete insulin and amylin, δ-cells synthesize somatostatin, and γ-cells release the pancreatic polypeptide [102]. Disturbance in the function of any of these cells due to the SARS-CoV-2 attack has specific outcomes. Lipase and amylase are digestive enzymes typically secreted into the duodenum by the acinar cells of the exocrine portion of the pancreas. Acinar and ductal cells exhibit high expression of ACE2, which may explain the mechanism of pancreatic enzyme elevation [103].

The uncontrollable systemic inflammatory response may exacerbate pancreatic injury. Pancreatic stellate cells activated during systemic inflammation release inflammatory chemokines and cytokines, inducing extracellular matrix deposition, and resulting in pancreatic fibrosis [104]. β-cell autoimmunity may happen by the recruitment of tissue-resident immune cells in response to systemic inflammation [102]. Upregulation of pro-inflammatory genes such as CXCL12 in the infected acinar cells occurs throughout SARS-CoV-2 infection. CXCL12 is strongly chemotactic for lymphocytes and leads to the migration of more immune cells to the tissue, and worsens the hyperinflammatory response [105].

Overall, acute pancreatitis during COVID-19 arises from the severe inflammatory response syndrome and the direct attack of SARS-CoV-2 on pancreatic cells. Other situations, such as hypoperfusion resulting from mechanical ventilation or shock in severe patients, might potentially contribute to pancreatic tissue injury [106].

## Conclusion

In addition to virus-induced cytopathic effects and inflammation, other processes, such as the association between the gut-lung axis and the microbiota, pre-existing GI diseases, and hypoxia may be involved in the GI tract in patients with COVID-19. Although liver damage during COVID-19 is relatively common, the exact underlying mechanisms are not clearly described yet. A direct attack by SARS-CoV-2, severe inflammatory response syndrome, hepatic ischemia-reperfusion injury, underlying liver disorders, and drug-related liver injury are all possible etiologic factors for liver damage in SARS-CoV-2 infection. Moreover, pancreatic injury is less common than liver injury; however, direct attack of SARS-CoV-2, uncontrollable systemic inflammatory reaction, and hypoperfusion may result in acute pancreatitis in severe COVID-19 patients. Further large population-based studies are needed to confirm the present findings.

## Conflict of Interest

The authors declare no conflict of interest.
